# Mechanical behavior analysis of fully grouted bolt under axial load

**DOI:** 10.1038/s41598-023-27673-6

**Published:** 2023-01-09

**Authors:** Xiujun Liu, Zhanguo Ma

**Affiliations:** 1 State Key Laboratory for Geomechanics and Deep Underground Engineering, China University of Mining and Technology, Xuzhou, 221116 China; 2 Shenzhen Geotechnical Investigation and Surveying Institute (Group) Co., Ltd., Shenzhen, 518028 China

**Keywords:** Natural hazards, Engineering

## Abstract

Based on the idea of discretization and the force balance analysis of each mass spring element, a spring-element analysis method for bolt is proposed. By analyzing the mechanical behavior of the bond interface of the fully grouted bolt, three coupling models of the bond interface, the slider model, the spring model and the spring-slider model, are proposed. Using the spring-element method, five load transfer models, namely the slider model, the spring model, the modified spring model, the spring-pulled slider model, and the spring-slider model, were deduced. And get the bolt displacement distribution function, axial force distribution function and shear stress distribution function under each model. The five proposed models are verified, analyzed and discussed by using the pull-out test of the smooth steel bolt and the threaded steel bolt. It is verified by experiments that this study is helpful to comprehensively understand the mechanical behavior of fully grouted bolts under axial load. The proposed spring element analysis method is simple and easy to understand and the results are reasonable.

## Introduction

Over the years, with the rapid development of geotechnical anchoring technology, bolts have also been widely used in reinforcement projects such as civil engineering and mining. Windsor^1^ defined that an integral reinforcement system consists of four fundamental elements: the rock, the reinforcing element, the external fixture and the internal fixture. At present, there are three main anchoring techniques widely used, they are: mechanical anchoring, grouting anchoring and friction anchoring. Among them, the grouting anchoring technology is the most popular in practice due to its advantages of easy installation and relatively low cost^2^. For a fully grouted bolt, the reinforcing element represent the steel bar, and the external fixture are the faceplate and nut. The bonding effect between the bolt and rock mainly depends on the internal fixture, which refers to the grout material, for instance, the resin or cement mortar for grouted bars. The bearing performance of fully grouted bolts is closely related to the type of the steel bar, grouting materials and formation lithology. In a fully grouted bolt system, the shear stress is generated at the bolt–grout interface and the grout–rock interface during the deformation of rock mass. The load can be transferred between bolts and rock mass via the shear resistance of the grout. An in-depth study of the bolt's load transfer mechanism helps us optimize the design of the bolt.

To understand the load transfer mechanism of the bolt, it can be realized by the methods such as field test, numerical simulation and theoretical analysis. In the field test of bolts, many scholars have carried out a lot of experimental research work^3–11^. These research results have laid a good foundation for the theoretical analysis of the fully grouted bolt. For the theoretical analysis of the fully grouted bolt’s load transfer mechanism, many scholars have also done the following typical works. Phillips^12^ and Farmer^3^ proposed a shear stress distribution at the bolt interface in the form of an exponential function. Scholars such as Wijk^13^ deduced the analytical solutions of the axial force and shear stress distributed along the bolt anchorage section in detail based on the displacement solution of Mindlin. Based on the assumption that the rock mass, the grout, the bolt and the interface between them are all in an elastic working state, Aydan et al.^14^ established the solution of the drawing load distribution of the bolt. Li and Stillborg^15^ developed three analysis models for rock bolts: one for bolts subjected to a concentrated pull load in pullout tests, one for bolts installed in uniformly deformed rock masses, and one for bolts subjected to the opening of individual rock joints. Based on the three-line shear-slip model of the anchorage interface, Ren et al.^2^ established an analytical solution for the axial force and shear stress distribution of the anchorage section under fully elastic, elastoplastic and fully plastic states. Using the nonlinear shear-slip model, Ma et al.^16^ preliminarily analyzed the load transfer and nonlinear characteristics of full-length bonded bolts under pull-out load. Based on the use of the trilinear model to consider the elasticity, softening and debonding behavior at the cable/grout interface, Chen et al.^17^ proposed an analysis model for fully grouted bolts under axial load. Li et al.^18^ proposed a novel constitutive model based on the modified Mohr–Coulomb failure criterion as well as a newly proposed non-linear dilation formulae for the development of governing equations to predict the mechanical behavior of different cable bolts under axial load. Based on the pull-out test results, Jahangir et al.^19^ proposed a new interface constitutive model for fully grouted rock-bolts and cable-bolts, and established a database to combine the published experimental data with in-house tests. However, the above-mentioned research work is not deep enough in the mechanical analysis of the bond interface of the fully grouted bolt. Moreover, in the bolts of different bonding mechanism types, the lack of analysis and comparison requires further research.

The purpose of this study is to analyze the mechanical behavior of the bond interface of fully grouted bolts. Based on the idea of bolt discretization and the force balance analysis of each mass-spring element, five load transfer analysis models, including the slider model, the spring model, the modified spring model, the spring-pulled slider model and the spring-slider model, are proposed. And use the bolt pull-out test done by Rong et al.^8^ to verify, analyze and discuss the above five analysis models. This study contributes to a comprehensive understanding of the mechanical behavior of fully grouted bolts under axial loads. The proposed spring element analysis method is simple and easy to understand and the results are reasonable.

## Discretization of bolt

**Figure 1:**
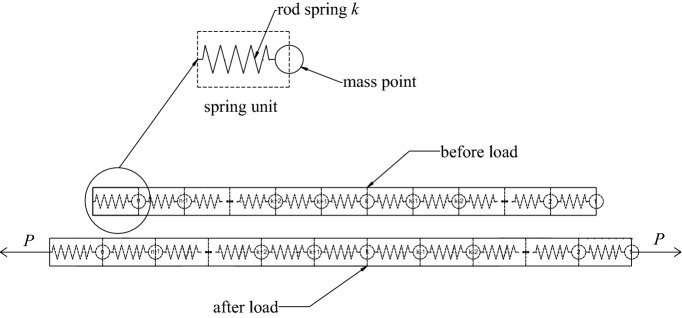
Discretization schematic diagram of free bar.

As shown in Fig. 1, a homogeneous free bar of equal cross-section, regardless of body force, can be discretized into *n* mass-point spring elements with the same stiffness *k* when no force is applied. The effect of this treatment is that each bar micro-segment is equivalent to a combination of a spring and an infinitesimal mass point. In the free state, its length is the same as that of the free bar micro-segment, and the external force on each bar micro-segment is concentrated on the mass point of the corresponding spring element. After the same tensile force *P* is applied at both ends, the bar is elongated by *s*, and the elongation Δ*s*_*i*_ of each spring element is *s*/*n*. From Hooke's law, we know:

1 $$\Delta s_{i} = \frac{Pl}{{nEA}},$$

where, *E* is the elastic modulus, *A* is the cross-sectional area, and *l* is the length of the bar.

Then the stiffness of each spring-element is:

2 $$k = \frac{nEA}{l}.$$

**Figure 2:**
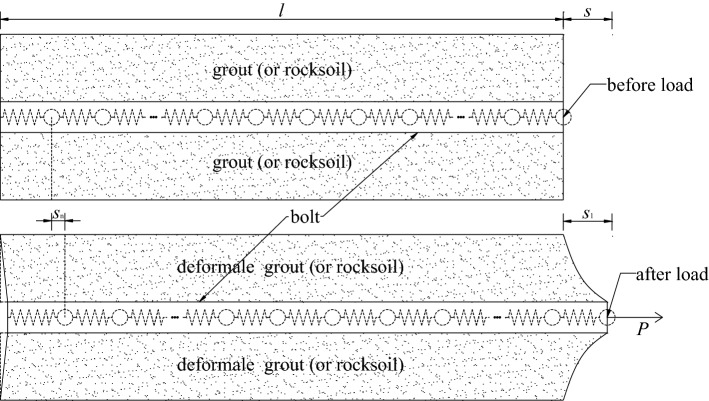
Discretization schematic diagram of bolt.

In the same way, as shown in Fig. 2, a homogeneous bolt of equal section can also be discretized into *n* spring-elements with the same stiffness *k* when no force is applied. After the tensile force *P* is applied to the top of the bolt, the top of the bolt produces a displacement *s*. Since the bolt is constrained by the side wall, the elongation Δ*s*_*i*_ of each spring element is not equal at this time, and:

3 $$\Delta s_{i} = \frac{{P_{i} }}{k},$$

where *P*_*i*_ is the spring tension of the *i*-th spring-element.

Starting from the top of the bolt, the spring-elements are numbered sequentially from 1 to *n*, and the displacements of the *i*-th spring-element and the *i* + 1-th spring-element are related as follows:

4 $$s_{i} - s_{i + 1} = \Delta s_{i} .$$

**Figure 3:**
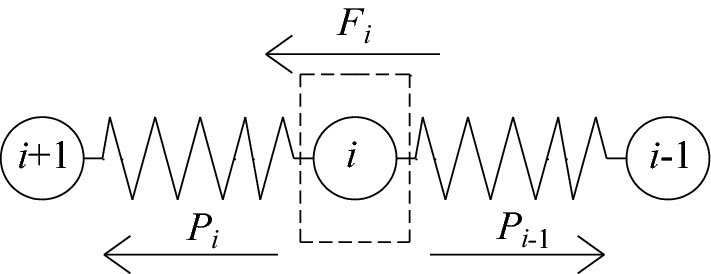
Force analysis diagram of the *i*-th spring-element.

Figure 3 is the force analysis diagram of the *i*-th spring-element. The spring-element *i* is not only subjected to the pulling forces *P*_*i*_ and *P*_*i*−1_ exerted by the adjacent spring-elements, but also the lateral resistance *F*_i_ provided by the side wall. It can be seen that:

5 $$F_{i} = P_{i - 1} - P_{i} .$$

Combining Eqs. (3) and (5), we can get:

6 $$\Delta s_{i - 1} - \Delta s_{i} = \frac{{F_{i} }}{k}.$$

Assuming *n* → ∞, when *i* varies from 1 to *n*, the distribution patterns of displacement *s*_*i*_, spring tension *P*_*i*_ and side wall resistance *F*_*i*_ of the *i*-th spring-element can be approximated as continuous distribution functions *s*(*x*), *P*(*x*) and *F*(*x*) along the bolt length, respectively. Among them, *x* is the length from the top of the bolt. Further, combined with Eq. (4), the first derivative of the displacement distribution function *s*(*x*) as $s^{\prime}(x) = \lim_{n \to \infty } {{ - \Delta s_{i} } \mathord{\left/ {\vphantom {{ - \Delta s_{i} } {({l \mathord{\left/ {\vphantom {l n}} \right. \kern-0pt} n})}}} \right. \kern-0pt} {({l \mathord{\left/ {\vphantom {l n}} \right. \kern-0pt} n})}}$, and the second derivative as $s^{\prime\prime}(x) = \lim_{n \to \infty } {{(\Delta s_{i - 1} - \Delta s_{i} )} \mathord{\left/ {\vphantom {{(\Delta s_{i - 1} - \Delta s_{i} )} {({l \mathord{\left/ {\vphantom {l n}} \right. \kern-0pt} n})}}} \right. \kern-0pt} {({l \mathord{\left/ {\vphantom {l n}} \right. \kern-0pt} n})}}^{2}$. Then Eqs. (3) and (6) can be respectively transformed into:

7a $$s^{\prime}(x) = - \frac{P(x)}{{k_{{\text{u}}} }};$$

7b $$s^{\prime\prime}(x) = \frac{F(x)}{{k_{{\text{u}}} }}.$$

In the formula, *k*_u_ is the stiffness of the bolt per unit length, namely *k*_u_ = *kl*/*n* = *EA*.

## Analysis of mechanical behavior of bolt interface

Fully grouted bolts can hold tensile, compressive, shear and bending loads, which leads to complex loading configurations in the field. In order to gain more insight into the load transfer mechanism between the bolt and the surrounding ground, it is necessary to evaluate the mechanical behavior of the bolt interface^19^. When a fully grouted bolt is subjected to a tensile load, the failure may occur either at the grout–rock interface, in the grout medium or at the bolt–grout interface, depending on which of the interfaces is the weakest^15^. Previous studies^9,20–25^ have shown that the bolt–grout interface is often more prone to failure than the grout-foundation interface, so the mechanical properties of the bolt–grout interface are the focus of their analysis. However, due to the difference and discontinuity of the media on both sides of the interface, for the entire grouted bolt, when one interface is damaged, the other interface will be deformed or damaged more or less, but the damage is relatively light. Therefore, from a rigorous point of view, the mechanical behavior of the two interfaces should be considered together.

**Figure 4:**
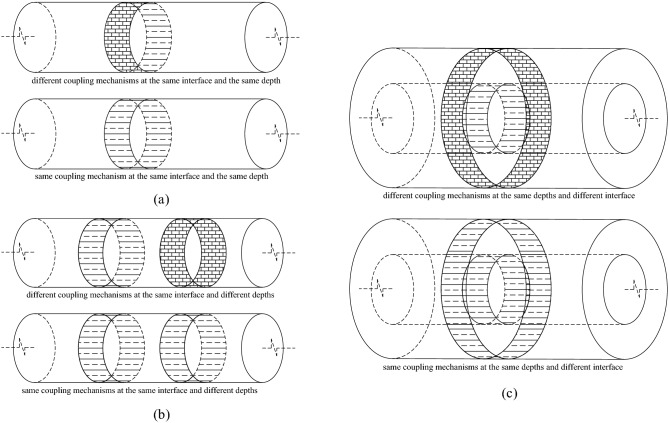
Interface coupling mechanism combination analysis diagram.

Considering the coupling mechanism of the interface, Windsor^1^ classified bolt reinforcement systems into three types: Continuous Mechanically Coupled (CMC), Continuous Frictionally Coupled (CFC) and Discretely Mechanically or Frictionally Coupled (DMFC). Many scholars^16,26–29^ believed that the fully grouted bolts belongs to the CMC system. According to Li and Stillborg^15^, the interface bond is provided by three mechanisms: adhesion, mechanical interlock and friction, and these mechanisms are lost progressively as debonding of the interface occurs. In fact, due to the heterogeneity of rocksoil and grout, as well as the interfacial gap caused by changes in temperature and humidity, the coupling characteristics on the bond interface of the bolt will also vary along the length of the bolt. Through a large number of experimental observations, it is found that for fully grouted bolts, there are at least two coupling mechanisms on the bolt–grout interface and the grout–rocksoil interface, which are mechanical interlock and friction, and there is no fixed sequence for the occurrence of the two coupling mechanisms. Singer^30^ pointed out that no adhesion exists in the bolt–resin interface and according to other studies of Yazici and Kaiser^31^ and Aziz and Webb^32^, adhesion strength has a small value which can be assumed to be negligible. For cement mortar bolts, although there may be adhesion between the interfaces, its effect is close to the mechanical interlock. Therefore, in this study, adhesion is classified as mechanical interlock. Therefore, for the entire grouted bolt, the following situations may occur. (i) At a certain depth of the bolt–grout interface or the grout–rocksoil interface, in the whole perimeter range, there may be all one kind of coupling, or two kinds of coupling may exist at the same time, as shown in Fig. 4a. (ii) At different depths of the bolt–grout interface or the grout–rocksoil interface, there may be all or not all of the same coupling mode, as shown in Fig. 4b. (iii) At a certain depth of the bolt, the bolt–grout interface and the grout–rocksoil interface may be in the same coupling mode, or may be in different coupling modes, as shown in Fig. 4c. (iv) Other more complicated combinations. Therefore, the interface coupling behavior of grouted bolts is a very complex system problem.

**Figure 5:**
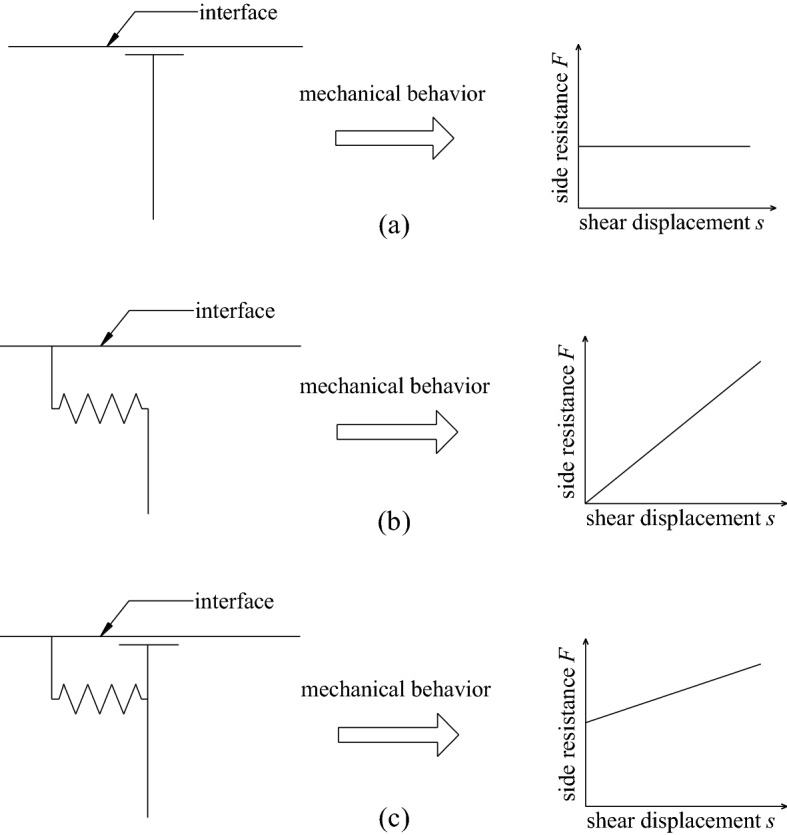
Models of interface coupling element. (**a**) Friction; (**b**) Mechanical interlock; (**c**) Combinatorial coupling.

Therefore, for the convenience of analysis, it is assumed that in a certain interface, if it can be determined that a certain coupling mode is dominant, the coupling behavior of the entire interface can be defined as this coupling mechanism. Otherwise, the coupling behavior of the entire interface can be defined as a combination of two coupling modes. For example, for the smooth steel bolt in the lower strength mortar, the coupling mechanism of the bolt–grout interface can be assumed to be friction, and the coupling element model is shown in Fig. 5a. For threaded steel bolts in higher-strength resin glue, the coupling mechanism of the bolt–grout interface can be assumed to be mechanical interlock, and the coupling element model is shown in Fig. 5b. For the cable bolt in the mortar with average strength, the coupling mechanism of the bolt–grout interface can be assumed to be a combination of friction and mechanical interlock. It can be called as combinatorial coupling, and the coupling element model is shown in Fig. 5c.

**Figure 6:**
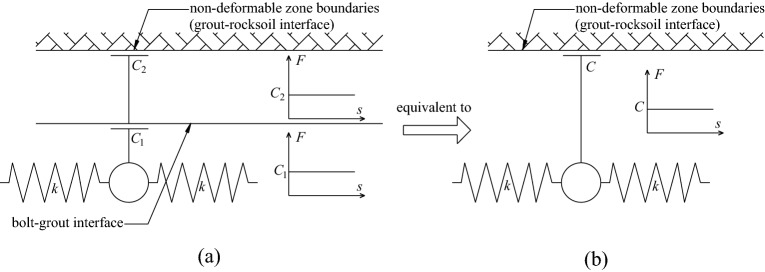
Evolution of the slider model. (**a**) When both interfaces are friction; (**b**) The slider model.

**Figure 7:**
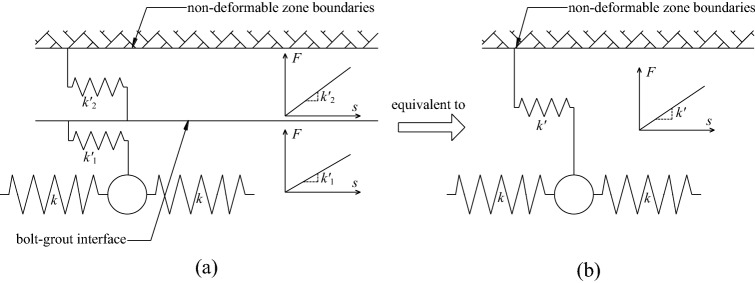
Evolution of the spring model. (**a**) When both interfaces are mechanical interlock; (**b**) The spring model.

**Figure 8:**
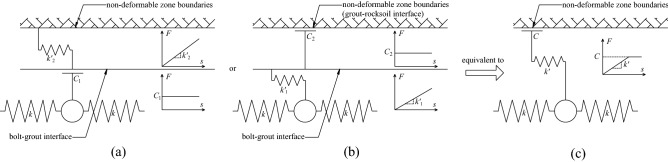
Evolution of the spring-pulled slider model. (**a**) When the bolt–grout interface is friction, and the grout–rocksoil interface is mechanical interlock; (**b**) When the bolt–grout interface is mechanical interlock, and the grout–rocksoil interface is friction; (**c**) The spring-pulled slider model.

**Figure 9:**
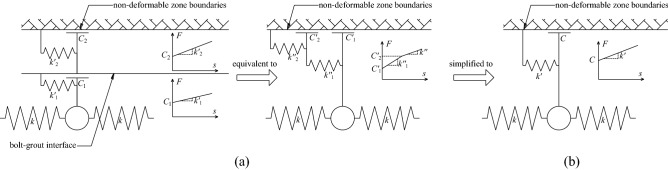
Evolution of the spring-slider model. (**a**) When the bolt–grout interface and the grout–rocksoil interface are both combinatorial coupling; (**b**) The spring-slider model.

**Figure 10:**
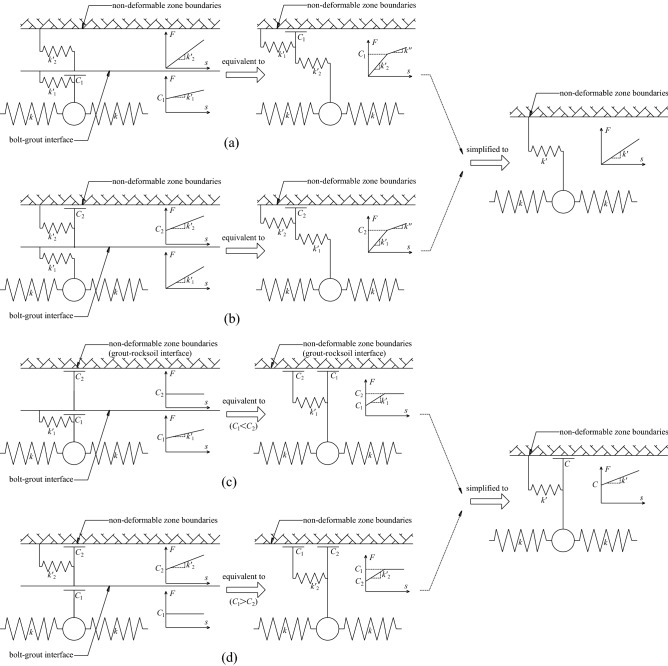
Several special cases. (**a**) When the bolt–grout interface is combinatorial coupling, and the grout–rocksoil interface is mechanical interlock; (**b**) When the bolt–grout interface is mechanical interlock, and the grout–rocksoil interface is combinatorial coupling; (**c**) When the bolt–grout interface is combinatorial coupling, and the grout–rocksoil interface is friction; (**d**) When the bolt–grout interface is friction, and the grout–rocksoil interface is combinatorial coupling.

When the mechanical behavior of the two interfaces of the grouted bolt is comprehensively analyzed, it is assumed that the deformation of the grout and the rocksoil is elastic. Combining the coupling behaviors on the two interfaces, the mechanical behavior of the medium (i.e.: grout, bolt–grout interface, grout–rocksoil interface, and rocksoil in deformation zone) between the bolt and the rocksoil in the non-deformed area can be simplified to the following models. (i) When both interfaces are friction, it can be equivalent to a slider model, as shown in Fig. 6b. (ii) When both interfaces are mechanical interlock, it can be equivalent to a spring model, as shown in Fig. 7b. (iii) When one of the two interfaces is friction and the other is mechanical interlock, it can be equivalent to a spring-pulled slider model, as shown in Fig. 8c. (iv) When both interfaces are the combinatorial coupling, it can be equivalent to a spring-slider model, as shown in Fig. 9b. In fact, the mechanical behavior at the interface is very complicated. In addition to the above situations, there are several coupling situations as shown in Fig. 10 on the two interfaces of the grouted bolt. However, because the models in these cases are too complex and are not conducive to calculation, they can be classified into the above-mentioned interface mechanics models after simplified processing.

In Fig. 6, *C*_1_ is the frictional resistance of the spring element on the bolt–grout interface, *C*_2_ is the frictional resistance on the grout–rocksoil interface allocated by the spring element, *C* is the comprehensive effect of *C*_1_ and *C*_2_. According to the Fig. 6, *C* is the smaller value of *C*_1_ and *C*_2_. However, due to the continuity of the grout and the rocksoil in the length direction of the bolt, this may not be the case in practice. This figure is just a simplified sketch for ease of understanding.

In Fig. 7, $k^{\prime}_{1}$ is the elastic stiffness coefficient of the grout corresponding to the spring-element, $k^{\prime}_{2}$ is the elastic stiffness coefficient of the rocksoil corresponding to the spring-element, *k*′ is the comprehensive effect of $k^{\prime}_{1}$ and$k^{\prime}_{2}$. According to the Fig. 7, *k*′ is the serial of $k^{\prime}_{1}$ and $k^{\prime}_{2}$. Similarly, due to the continuity of the grout and the rocksoil in the length direction of the bolt, this may not be the case in practice also. This figure is also a simplified sketch for ease of understanding.

Figure 8 shows that *C* and *k*′ in Fig. 8c correspond to *C*_1_ and $k^{\prime}_{2}$ in Fig. 8a, and *C*_2_ and $k^{\prime}_{1}$ in Fig. 8b, respectively. However, due to the continuity of the grout and the rocksoil in the length direction of the bolt, the actual situation may not be the case, and this figure is only a schematic diagram for the convenience of understanding.

In Fig. 9a, $C^{\prime}_{1}$ is the smaller value of *C*_1_ and *C*_2_, $k^{\prime\prime}_{1}$ is the elastic stiffness coefficient on the interface where the small value is located, $C^{\prime}_{2}$ and $k^{\prime\prime}_{2}$ are the frictional resistance and elastic stiffness coefficient on the other interface, respectively, and *k*″ is the elastic stiffness coefficient of $k^{\prime}_{1}$ and $k^{\prime}_{2}$ in series. The *C* and *k*′ in Fig. 9b are the results of the synthesis of *C*_1_, *C*_2_ and $k^{\prime}_{1}$, $k^{\prime}_{2}$, respectively. Due to some simplifications, the relationship between them is not clear, which can be obtained by the interface mechanical test.

In Fig. 10, *k*″ is the elastic stiffness coefficient of $k^{\prime}_{1}$ and $k^{\prime}_{2}$ connected in series. Similarly, *C* and *k*′ are the results of the synthesis of *C*_1_, *C*_2_ and $k^{\prime}_{1}$, $k^{\prime}_{2}$, respectively. Due to some simplifications, the relationship between them is also unclear, which can be obtained through the interface mechanical test. It can be clearly seen that when *C*_1_ ≥ *C*_2_ in Fig. 10c, and when *C*_1_ ≤ *C*_2_ in Fig. 10d, the interface mechanical models under the two interface coupling conditions are both degenerated into the slider model.

**Figure 11:**
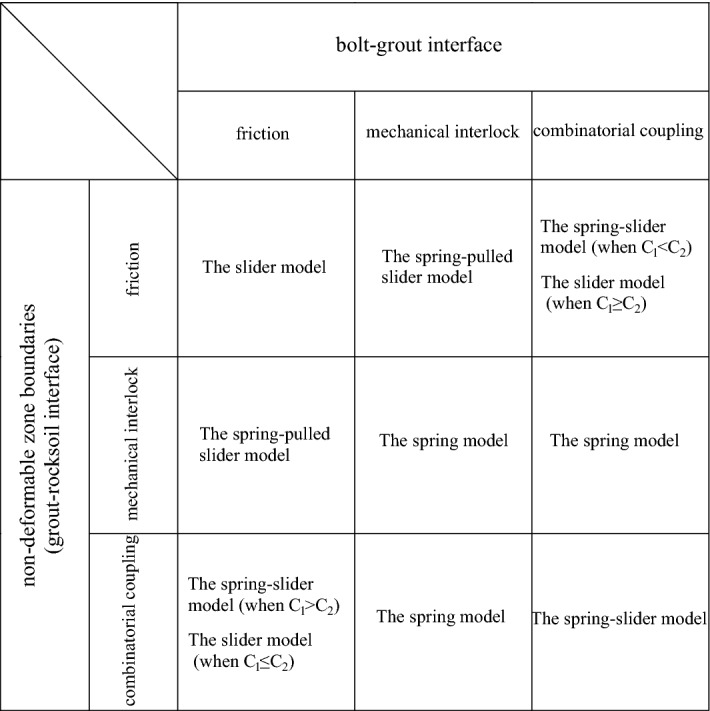
Summary of interface coupling condition analysis.

To sum up, considering that there are three coupling modes of friction, mechanical interlock, and friction-mechanical interlock combination on the bolt–grout interface and the grout–rocksoil interface, respectively, nine interface coupling conditions can be obtained. The nine interface coupling conditions can be classified into four mechanical analysis models, namely the slider model, the spring model, the spring-pulled slider model, and the spring-slider model, as shown in Fig. 11.

## Model analysis

### Slider model

**Figure 12:**
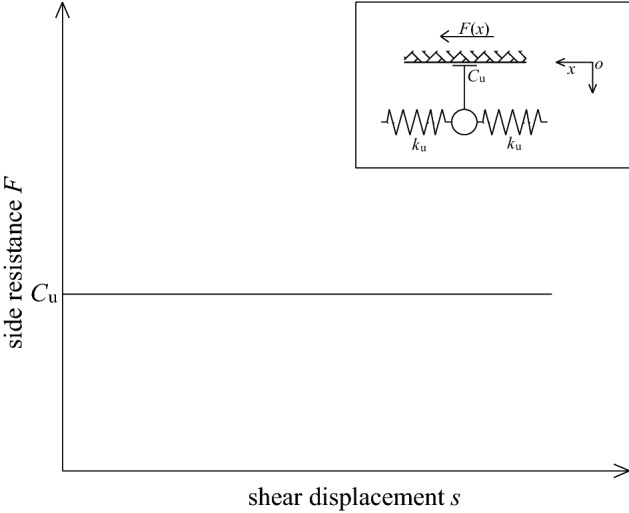
Schematic diagram of slider model.

As shown in Fig. 12, the slider model assumes that the lateral resistance provided by the sidewall to the bolt is a constant value, namely:

8 $$F(x) = C_{{\text{u}}} ,$$

where *C*_u_ is the lateral resistance per unit bolt length, namely *C*_u_ = *C*/(*l*/*n*).

Substituting Eq. (8) into Eq. (7), we can get:

9 $$s^{\prime\prime}(x) - \frac{{C_{{\text{u}}} }}{{k_{{\text{u}}} }} = 0.$$

The general solution to the above equation is:

10 $$s(x) = \frac{{C_{{\text{u}}} }}{{2k_{{\text{u}}} }}x^{2} + {\text{A}}_{1} x + {\text{A}}_{2} ,$$

where A_1_ and A_2_ are the parameters to be sought.

It is known that the boundary conditions are:

11a $$s(x)\left| {_{x = 0} } \right. = s_{0} ;$$

11b $$s^{\prime}(x)\left| {_{x = 0} } \right. = - \frac{{P_{0} }}{{k_{{\text{u}}} }};$$

11c $$s^{\prime}(x)\left| {_{x = l} } \right. = 0,$$

where *s*_0_ and *P*_0_ are the displacement and pull-out force at the top of the bolt, respectively.

Substituting boundary condition Eqs. (11a)–(11c) into Eq. (10), we can obtain: $C_{{\text{u}}} = P_{0} /l$; $A_{1} = - P_{0} /k_{{\text{u}}}$; ${\text{A}}_{2} = s_{0}$.

Then the displacement distribution function of the bolt can be obtained as:

12 $$s(x) = \frac{{P_{0} }}{{2k_{{\text{u}}} l}}x^{2} - \frac{{P_{0} }}{{k_{{\text{u}}} }}x + s_{0} .$$

Taking the derivative of *x* on both sides of Eq. (12), and substituting *s*′(*x*) into Eq. (7a), the axial force distribution function of the bolt is obtained as:

13 $$P(x) = P_{0} \left( {1 - \frac{x}{l}} \right).$$

Then the shear stress distribution function of the bolt as:

14 $$\tau (x) = \frac{{P_{0} }}{{2\pi r_{{\text{b}}} l}},$$

where *r*_b_ is the radius of the bolt.

Obviously, under the slider model, the pull-out force at the top of the bolt as *P*_0_ = *C*_u_*l*, and *P*_0_ increases linearly with *C*_u_. The model assumes that the lateral friction resistance of the bolt is constant. However, according to a large number of field tests, it is found that almost all fully grouted bolts do not meet this assumption. Because the slider model has the advantages of simplicity and ease of use, this model is more suitable for fully grouted bolts with low interfacial bond strength, short length, and mainly rely on friction to provide pull-out resistance, such as short smooth steel bolt. The analytical accuracy of the model depends on a reasonable value of *C*_u_.

### Spring model

**Figure 13:**
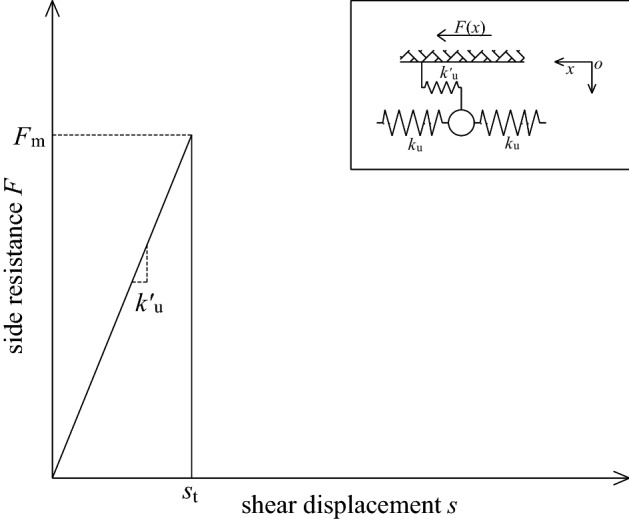
Schematic diagram of spring model.

As shown in Fig. 13, spring model assumes that the lateral resistance provided by the sidewall to the bolt first increases linearly with the displacement of the spring-element. When the displacement *s* > *s*_t_, the sidewall spring is pulled off, and its side resistance is directly reduced from the ultimate value *F*_m_ to 0. From this, the lateral resistance can be divided into two stages: before and after the spring breaks, as follows:

15 $$F(x) = \left\{ {\begin{array}{*{20}l} {k_{{\text{u}}}^{{\prime}} s(x)} \hfill & {\quad (0 < s \le s_{t} )} \hfill \\ 0 \hfill & {\quad (s_{t} < s \le s_{0} )} \hfill \\ \end{array} } \right..$$

where, *s*_t_ is the ultimate displacement of the sidewall spring, and $s_{{\text{t}}} = {{F_{{\text{m}}} } \mathord{\left/ {\vphantom {{F_{{\text{m}}} } {k_{{\text{u}}}^{{\prime}} }}} \right. \kern-0pt} {k_{{\text{u}}}^{{\prime}} }}$.

#### Before the sidewall spring is broken (0 < *s* ≤ *s*_t_)

Substituting Eq. (15) into Eq. (7b), the bolt load transfer equation at this time can be obtained as:

16 $$s^{\prime\prime}(x) - \frac{{k_{{\text{u}}}^{{\prime}} }}{{k_{{\text{u}}} }}s(x) = 0.$$

The general solution to the above equation as:

17 $$s(x) = {\text{B}}_{1} {\text{e}}^{\lambda x} + {\text{B}}_{2} {\text{e}}^{ - \lambda x} .$$

where, B_1_ and B_2_ are the parameters to be sought, and $\lambda =\sqrt{{k}_{\mathrm{u}}^{{\prime}}/{k}_{\mathrm{u}}}$.

Substituting boundary condition Eqs. (11a)–(11c) into Eq. (17), we can obtain:

$${\text{B}}_{1} = s_{0} \frac{{{\text{e}}^{ - \lambda l} }}{{{\text{e}}^{\lambda l} + {\text{e}}^{ - \lambda l} }} = \frac{{P_{0} }}{{\lambda k_{{\text{u}}} }} \cdot \frac{{{\text{e}}^{ - \lambda l} }}{{{\text{e}}^{\lambda l} - {\text{e}}^{ - \lambda l} }};\quad {\text{B}}_{2} = s_{0} \frac{{{\text{e}}^{\lambda l} }}{{{\text{e}}^{\lambda l} + {\text{e}}^{ - \lambda l} }} = \frac{{P_{0} }}{{\lambda k_{{\text{u}}} }} \cdot \frac{{{\text{e}}^{\lambda l} }}{{{\text{e}}^{\lambda l} - {\text{e}}^{ - \lambda l} }}.$$

Then the displacement distribution function of the bolt can be obtained as:

18 $$s(x) = s_{0} \frac{\cosh [\lambda (l - x)]}{{\cosh (\lambda l)}} = \frac{{P_{0} }}{{\lambda k_{{\text{u}}} }} \cdot \frac{\cosh [\lambda (l - x)]}{{\sinh (\lambda l)}}.$$

Taking the derivative of *x* on both sides of Eq. (18), and substituting *s*′(*x*) into Eq. (7a), the axial force distribution function of the bolt can obtained as:

19 $$P(x) = \lambda k_{{\text{u}}} s_{0} \frac{\sinh [\lambda (l - x)]}{{\cosh (\lambda l)}} = P_{0} \frac{\sinh [\lambda (l - x)]}{{\sinh (\lambda l)}}.$$

Substitute Eq. (18) into Eq. (15) to obtain the shear stress distribution function of the bolt as:

20 $$\tau (x) = \frac{{k_{{\text{u}}}^{{\prime}} s_{0} }}{{2{\uppi }r_{{\text{b}}} }} \cdot \frac{\cosh [\lambda (l - x)]}{{\cosh (\lambda l)}} = \frac{{P_{0} \lambda }}{{2{\uppi }r_{{\text{b}}} }} \cdot \frac{\cosh [\lambda (l - x)]}{{\sinh (\lambda l)}}.$$

#### After the sidewall spring is broken (*s*_t_ < *s* ≤ *s*_0_)

Assuming that the sidewall spring at a certain depth *x*_t_ is just in the critical state of being pulled off, then the displacement of the spring-element at that location is just as *s*_t_, and it can be known that the sidewall springs within the depth of *x*_t_ are all pulled off. Thereby, the bolt can be divided into the sidewall spring breaking areas and the non-breaking areas. At this point, a new boundary condition is added at *x*_t_ as follows:

21a $$s(x)\left| {_{{x = x_{{\text{t}}} }} } \right. = s_{{\text{t}}} ;$$

21b $$s^{{\prime}} (x)\left| {_{{x = x_{{\text{t}}} }} } \right. = - \frac{{P_{0} }}{{k_{{\text{u}}} }}.$$

Substituting the boundary conditions at this time into Eq. (17), the coefficients can be obtained as:

$${\text{B}}_{1} = s_{{\text{t}}} \frac{{{\text{e}}^{ - \lambda l} }}{{{\text{e}}^{{\lambda (l - x_{{\text{t}}} )}} + {\text{e}}^{{ - \lambda (l - x_{{\text{t}}} )}} }};{\kern 1pt} \quad {\text{B}}_{2} = s_{{\text{t}}} \frac{{{\text{e}}^{\lambda l} }}{{{\text{e}}^{{\lambda (l - x_{{\text{t}}} )}} + {\text{e}}^{{ - \lambda (l - x_{{\text{t}}} )}} }},\quad {\text{and}}\quad x_{{\text{t}}} = \frac{{k_{{\text{u}}} }}{{P_{0} }}(s_{{0}} - s_{{\text{t}}} ).$$

Then the displacement distribution function of the bolt can be obtained as:

22 $$s(x) = \left\{ {\begin{array}{*{20}l} {s_{{\text{t}}} \frac{\cosh [\lambda (l - x)]}{{\cosh [\lambda (l - x_{{\text{t}}} )]}}} \hfill & {\quad (x_{{\text{t}}} < x \le l)} \hfill \\ {s_{{0}} - \frac{{P_{0} }}{{k_{{\text{u}}} }}x} \hfill & {\quad (0 \le x \le x_{{\text{t}}} )} \hfill \\ \end{array} } \right.,$$

the axial force distribution function of the bolt as:

23 $$P(x) = \left\{ {\begin{array}{*{20}l} {\lambda k_{{\text{u}}} s_{{\text{t}}} \frac{\sinh [\lambda (l - x)]}{{\cosh [\lambda (l - x_{{\text{t}}} )]}}} \hfill & {\quad (x_{{\text{t}}} < x \le l)} \hfill \\ {P_{0} } \hfill & {\quad (0 \le x \le x_{{\text{t}}} )} \hfill \\ \end{array} } \right.,$$

and the shear stress distribution function of the bolt as:

24 $$\tau (x) = \left\{ {\begin{array}{*{20}l} {\frac{{F_{{\text{m}}} }}{{2{\uppi }r_{{\text{b}}} }} \cdot \frac{\cosh [\lambda (l - x)]}{{\cosh [\lambda (l - x_{{\text{t}}} )]}}} \hfill & {\quad (x_{{\text{t}}} < x \le l)} \hfill \\ 0 \hfill & {\quad (0 \le x \le x_{{\text{t}}} )} \hfill \\ \end{array} } \right..$$

According to the continuity of *P*(*x*) at *x* = *x*_t_, the pull-out force at the top of the bolt can be obtained as:

25 $$P_{0} = \lambda k_{{\text{u}}} s_{{\text{t}}} \tanh [\lambda (l - x_{{\text{t}}} )].$$

It can be seen from Eq. (25) that *P*_0_ changes with the change of *x*_t_, and the pull-out force at the top_t_ of the bolt is a function *P*_0_(*x*). After differentiating it, we get $P_{0}^{{\prime}} (x_{{\text{t}}} ) = - F_{{\text{m}}} {{\{ 1 - {\text{tanh}}}}^{2} {[}\lambda {(}l{ - }x_{{\text{t}}} {{)]\} < }}0$. It can be seen that *P*_0_ decreases with the increase of *x*_t_, and the ultimate pull-out force at the top of the bolt as:

26 $$P_{0\max } = \frac{{F_{{\text{m}}} }}{\lambda }\tanh (\lambda l).$$

In this case, the critical failure depth of the shear plane as $x_{{{\text{tj}}}} = 0$.

It can be seen from the above analysis that the spring model is more suitable for fully grouted bolts with high interface bond strength or strong mechanical coupling, and there is almost no frictional resistance after the debonding of the bolt interface. However, according to a large number of experimental studies, this situation is difficult to satisfy. Therefore, the model has a very small scope of applicability.

### Modified spring model

**Figure 14:**
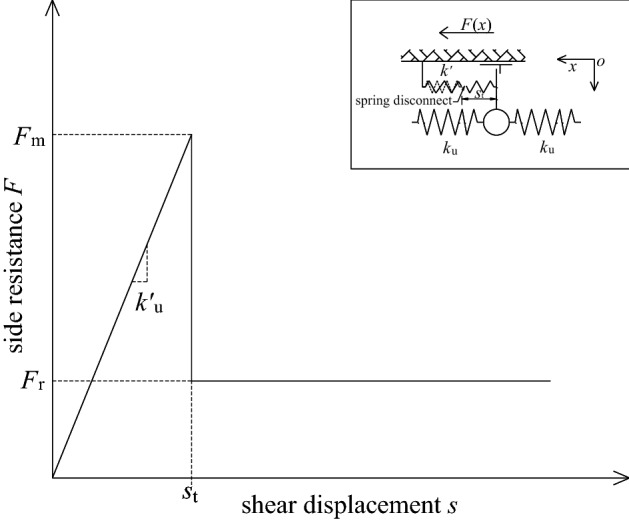
Schematic diagram of modified spring model.

According to the actual situation of the project, when the bolt interface is damaged, a part of the friction resistance remains on the damaged interface. Therefore, the assumptions of the spring model are inconsistent with the actual engineering and need to be corrected. It is assumed that after the sidewall spring is broken, the side resistance of the damaged interface is directly reduced from the ultimate value *F*_m_ to the residual friction resistance *F*_r_ and remains unchanged, as shown in Fig. 14. Therefore, the side resistance can also be divided into two stages before and after the spring is broken, as follows:

27 $$F(x) = \left\{ {\begin{array}{*{20}l} {k_{{\text{u}}}^{{\prime}} s(x)} \hfill & {\quad (0 < s \le s_{{\text{t}}} )} \hfill \\ {F_{{\text{r}}} } \hfill & {\quad (s_{{\text{t}}} < s \le s_{0} )} \hfill \\ \end{array} } \right..$$

Obviously, the model is exactly the same as the spring model before the sidewall spring is broken, and the difference is the mechanical behavior after the sidewall spring is broken. According to the previous derivation, the bolt load transfer equation at this time as:

28 $$s(x) = \left\{ {\begin{array}{*{20}l} {{\text{B}}_{1} {\text{e}}^{\lambda x} + {\text{B}}_{2} {\text{e}}^{ - \lambda x} } \hfill & {\quad (x_{{\text{t}}} < x \le l)} \hfill \\ {\frac{{F_{{\text{r}}} }}{{2k_{{\text{u}}} }}x^{2} + {\text{A}}_{1} x + {\text{A}}_{2} } \hfill & {\quad (0 \le x \le x_{{\text{t}}} )} \hfill \\ \end{array} } \right..$$

Substituting the boundary conditions into Eq. (28), the coefficients at this time can be obtained as:

$${\text{A}}_{1} = - \frac{{P_{0} }}{{k_{{\text{u}}} }};\quad {\text{A}}_{2} = s_{0} ;\quad {\text{B}}_{1} = s_{{\text{t}}} \frac{{{\text{e}}^{ - \lambda l} }}{{{\text{e}}^{{\lambda (l - x_{{\text{t}}} )}} + {\text{e}}^{{ - \lambda (l - x_{{\text{t}}} )}} }};\quad {\text{B}}_{2} = s_{{\text{t}}} \frac{{{\text{e}}^{\lambda l} }}{{{\text{e}}^{{\lambda (l - x_{{\text{t}}} )}} + {\text{e}}^{{ - \lambda (l - x_{{\text{t}}} )}} }}.$$

Thus, it can be obtained as:

29a $$s(x) = \left\{ {\begin{array}{*{20}l} {s_{{\text{t}}} \frac{\cosh [\lambda (l - x)]}{{\cosh [\lambda (l - x_{{\text{t}}} )]}}} \hfill & {\quad (x_{{\text{t}}} < x \le l)} \hfill \\ {\frac{{F_{{\text{r}}} }}{{2k_{{\text{u}}} }}x^{2} - \frac{{P_{0} }}{{k_{{\text{u}}} }}x + s_{0} } \hfill & {\quad (0 \le x \le x_{{\text{t}}} )} \hfill \\ \end{array} } \right.;$$

29b $$P(x) = \left\{ {\begin{array}{*{20}l} {\lambda k_{{\text{u}}} s_{{\text{t}}} \frac{\sinh [\lambda (l - x)]}{{\cosh [\lambda (l - x_{{\text{t}}} )]}}} \hfill & {\quad (x_{{\text{t}}} < x \le l)} \hfill \\ {P_{0} - F_{{\text{r}}} x} \hfill & {\quad (0 \le x \le x_{{\text{t}}} )} \hfill \\ \end{array} } \right.;$$

29c $$\tau (x) = \left\{ {\begin{array}{*{20}l} {\frac{{F_{{\text{m}}} }}{{2{\uppi }r_{{\text{b}}} }} \cdot \frac{\cosh [\lambda (l - x)]}{{\cosh [\lambda (l - x_{{\text{t}}} )]}}} \hfill & {\quad (x_{{\text{t}}} < x \le l)} \hfill \\ {\frac{{F_{{\text{r}}} }}{{2{\uppi }r_{{\text{b}}} }}} \hfill & {\quad (0 \le x \le x_{{\text{t}}} )} \hfill \\ \end{array} } \right..$$

According to the continuity of *P*(*x*) at *x* = *x*_t_, the pull-out force at the top of the bolt can be obtained as:

30 $$P_{0} = \lambda k_{{\text{u}}} s_{{\text{t}}} \tanh [\lambda (l - x_{{\text{t}}} )] + F_{{\text{r}}} x_{{\text{t}}} ,$$

and solving the above equation, *x*_t_ can be obtained.

In the same way, the first-order derivative of the pull-out force function *P*_0_(*x*_t_) at the top of the bolt can be obtained as $P_{0}^{{\prime}} (x_{{\text{t}}} ) = F_{{\text{r}}} - F_{{\text{m}}} {{\{ 1 - {\text{tanh}}}}^{2} {[}\lambda {(}l{ - }x_{{\text{t}}} {{)]\} }}$. It can be found by observation that when *x*_t_ increases from 0 to *l*, since *F*_r_ < *F*_m_, $P^{\prime}_{0}$(*x*_t_) first changes from a positive value to 0, and then from 0 to a negative value. Therefore, it can be known that *P*_0_(*x*_t_) is a convex function and has a maximum value. Let $P^{\prime}_{0}$(*x*_t_) = 0, the critical failure depth of the shear plane under the ultimate drawing force *P*_0max_ can be obtained as:

31 $$x_{{{\text{tj}}}} = l - \frac{1}{2\lambda }\ln \frac{{1 + \sqrt {1 - \alpha } }}{{1 - \sqrt {1 - \alpha } }},$$

where $$\alpha = {{F_{{\text{r}}} } \mathord{\left/ {\vphantom {{F_{{\text{r}}} } {F_{{\text{m}}} }}} \right. \kern-0pt} {F_{{\text{m}}} }}$$.

At this time, the ultimate pull-out force as:

32 $$P_{0\max } = \frac{{F_{{\text{m}}} }}{\lambda }\tanh\left( {\frac{1}{2}\ln \frac{{1 + \sqrt {1 - \alpha } }}{{1 - \sqrt {1 - \alpha } }}} \right) + F_{{\text{r}}} \left( {l - \frac{1}{2\lambda }\ln \frac{{1 + \sqrt {1 - \alpha } }}{{1 - \sqrt {1 - \alpha } }}} \right).$$

According to the continuity of *s*(*x*) at *x* = *x*_t_, the top displacement of the bolt after the sidewall spring is broken can be obtained as:

33 $$s_{0} = s_{{\text{t}}} - \frac{{F_{{\text{r}}} x_{{\text{t}}}^{{2}} }}{{2k_{{\text{u}}} }} + \frac{{P_{0} x_{{\text{t}}} }}{{k_{{\text{u}}} }}.$$

It is obvious that the modified spring model is also suitable for fully grouted bolts with high interfacial bonding strength or strong mechanical coupling. Since there is more or less a certain frictional resistance after the debonding of the bonding interface, this model has a wider application range than the spring model. The modified spring model is applicable to almost all fully grouted bolts except for special bolts that rely entirely or almost entirely on friction to provide pull-out resistance.

### Spring-pulled slider model

**Figure 15:**
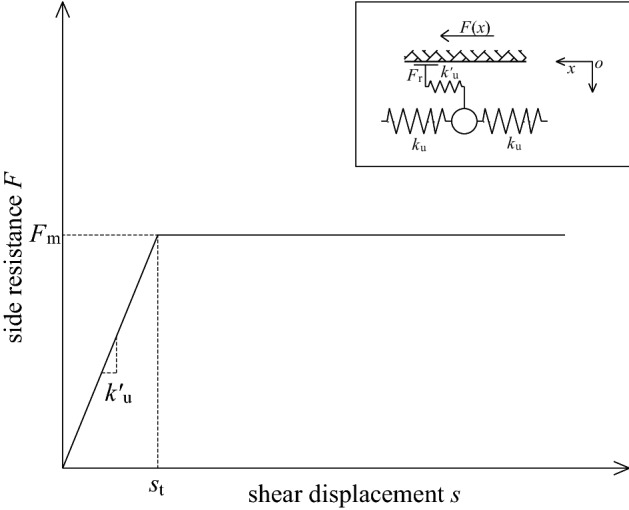
Schematic diagram of spring-pulled slider model.

As shown in Fig. 15, the model assumes that the sidewall springs are connected to the hole wall by a slider. The side resistance first increases linearly with the displacement of the spring-element, and the slider is pulled when the side resistance increases to a certain extent. At this time, the side resistance reaches the ultimate value *F*_m_, which is the same as the sliding friction resistance, and remains unchanged with the increase of displacement. In fact, this model is very similar to the modified spring model, but it is just a special case of the modified spring model when *α* = 1. It can be seen that the critical failure depth of the shear plane calculated by this model is *x*_tj_ = *l*, and the ultimate pull-out force is *P*_0max_ = *F*_m_*l*.

Since the spring-pulled slider model is a special case of the modified spring model, its scope of application is also limited. The model assumes that the side friction resistance after debonding of the bonding interface is the limit side friction resistance and remains unchanged. Therefore, this model is suitable for fully grouted bolts with low interfacial bond strength, and the interface after debonding still has high friction resistance. Moreover, the model also runs the risk of overestimating the ultimate bearing capacity of fully grouted bolts.

### Spring-slider model

**Figure 16:**
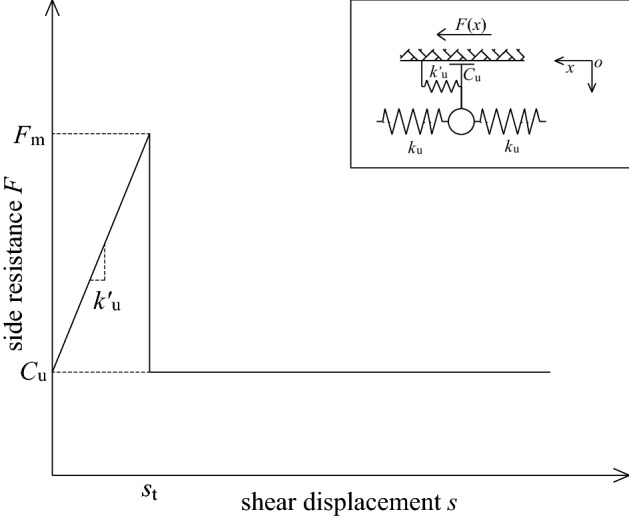
Schematic diagram of spring-slider model.

As shown in Fig. 16, this model assumes that the lateral resistance provided by the sidewall to the bolt consists of two parts: one part is a constant value *C*_u_, and the other part increases linearly with the displacement of the spring-element. When the displacement *s* > *s*_t_, the sidewall spring is pulled off, and its side resistance is directly reduced from the ultimate value *F*_m_ to *C*_u_. From this, the side resistance can also be divided into two stages before and after the spring breaks, as follows:

34 $$F(x) = \left\{ {\begin{array}{*{20}l} {k_{{\text{u}}}^{{\prime}} s(x) + C_{{\text{u}}} } \hfill & {\quad (0 < s \le s_{{\text{t}}} )} \hfill \\ {C_{{\text{u}}} } \hfill & {\quad (s_{{\text{t}}} < s \le s_{0} )} \hfill \\ \end{array} } \right.,$$

in which $$s_{{\text{t}}} = {{(F_{{\text{m}}} - C_{{\text{u}}} )} \mathord{\left/ {\vphantom {{(F_{{\text{m}}} - C_{{\text{u}}} )} {k_{{\text{u}}}^{{\prime}} }}} \right. \kern-0pt} {k_{{\text{u}}}^{{\prime}} }}$$.

#### Before the sidewall spring is broken (0 < *s* ≤ *s*_t_)

Substitute Eq. (34) into Eq. (7b) to obtain the bolt load transfer equation at this time as:

35 $$s^{{\prime\prime}} (x) - \frac{{k_{{\text{u}}}^{{\prime}} }}{{k_{{\text{u}}} }}s(x) - \frac{{C_{{\text{u}}} }}{{k_{{\text{u}}} }} = 0.$$

The general solution of Eq. (35) is:

36 $$s(x) = {\text{B}}_{1} {\text{e}}^{\lambda x} + {\text{B}}_{2} {\text{e}}^{ - \lambda x} - \frac{{C_{{\text{u}}} }}{{k_{{\text{u}}}^{{\prime}} }}.$$

Substituting the boundary condition Eqs. (11a)–(11c) into Eq. (36), it can be obtained:

$${\text{B}}_{1} = \left( {s_{0} + \frac{{C_{{\text{u}}} }}{{k_{{\text{u}}}^{{\prime}} }}} \right)\frac{{{\text{e}}^{ - \lambda l} }}{{{\text{e}}^{\lambda l} + {\text{e}}^{ - \lambda l} }};\quad {\text{B}}_{2} = \left( {s_{0} + \frac{{C_{{\text{u}}} }}{{k_{{\text{u}}}^{{\prime}} }}} \right)\frac{{{\text{e}}^{\lambda l} }}{{{\text{e}}^{\lambda l} + {\text{e}}^{ - \lambda l} }};\quad C_{{\text{u}}} = k_{{\text{u}}}^{{\prime}} \left[ {\frac{{P_{{0}} }}{{\lambda k_{{\text{u}}} }} \cdot \frac{\cosh (\lambda l)}{{\sinh (\lambda l)}} - s_{0} } \right] = \frac{{P_{{0}} \lambda }}{\tanh (\lambda l)} - k_{{\text{u}}}^{{\prime}} s_{0} .$$

Then the displacement distribution function of the bolt can be obtained as:

37 $$s(x) = \frac{{P_{0} }}{{\lambda k_{{\text{u}}} }} \cdot \frac{\cosh [\lambda (l - x)] - \cosh (\lambda l)}{{\sinh (\lambda l)}} + s_{0} .$$

Taking the derivative of *x* on both sides of Eq. (37), and substituting *s*′(*x*) into Eq. (7a), the axial force distribution function of the bolt can be obtained as:

38 $$P(x) = P_{0} \frac{\sinh [\lambda (l - x)]}{{\sinh (\lambda l)}}.$$

Substitute Eq. (37) into Eq. (34) to obtain the shear stress distribution function of the bolt as:

39 $$\tau (x) = \frac{{P_{0} \lambda }}{{2{\uppi }r_{{\text{b}}} }} \cdot \frac{\cosh [\lambda (l - x)]}{{\sinh (\lambda l)}}.$$

By observing the calculation formula of *C*_u_, it can be seen that its value varies with *P*_0_ and *s*_0_, which are known. In order to facilitate the calculation, take the average value $\overline{C}_{{\text{u}}}$ of *C*_u_ calculated under various loads before the side wall spring is broken, and substitute $\overline{C}_{{\text{u}}}$ into the formula after the side wall spring is broken for calculation. In addition, when *s*_0_ = *s*_t_, substituting *s*_t_ into the calculation formula of *C*_u_, the pull-out force of the sidewall spring at the top of the bolt when it is in the critical state of being pulled off can be obtained, that is, the elastic limit pull-out force:

40 $$P_{{0{\text{e}}\max }} = \frac{{F_{{\text{m}}} }}{\lambda }\tanh (\lambda l).$$

#### After the sidewall spring is broken (*s*_t_ < *s* ≤ *s*_0_)

Similarly, assuming that the sidewall spring at a certain depth *x*_t_ is just in the critical state of being pulled off, then the displacement of the spring-element at that position is just *s*_t_, and it can be seen that the sidewall springs within the depth *x*_t_ are all pulled off. Thereby, the bolt can also be divided into the sidewall spring breaking areas and the non-breaking areas. According to the previous derivation, the load transfer equation of the bolt at this time as:

41 $$s(x) = \left\{ {\begin{array}{*{20}l} {{\text{B}}_{1} {\text{e}}^{\lambda x} + {\text{B}}_{2} {\text{e}}^{ - \lambda x} - \frac{{\overline{C}_{{\text{u}}} }}{{k_{{\text{u}}}^{{\prime}} }}} \hfill & {\quad (x_{{\text{t}}} < x \le l)} \hfill \\ {\frac{{\overline{C}_{{\text{u}}} }}{{2k_{{\text{u}}} }}x^{2} + {\text{A}}_{1} x + {\text{A}}_{2} } \hfill & {\quad (0 \le x \le x_{{\text{t}}} )} \hfill \\ \end{array} } \right..$$

Substituting the boundary conditions at this time into Eq. (41), the coefficients can be obtained as:

$${\text{A}}_{1} = - \frac{{P_{0} }}{{k_{{\text{u}}} }};\quad {\text{A}}_{2} = s_{0} ;\quad {\text{B}}_{1} = \frac{{F_{{\text{m}}} }}{{k_{{\text{u}}}^{{\prime}} }} \cdot \frac{{{\text{e}}^{ - \lambda l} }}{{{\text{e}}^{{\lambda (l - x_{{\text{t}}} )}} + {\text{e}}^{{ - \lambda (l - x_{{\text{t}}} )}} }};\quad {\text{B}}_{2} = \frac{{F_{{\text{m}}} }}{{k_{{\text{u}}}^{{\prime}} }} \cdot \frac{{{\text{e}}^{\lambda l} }}{{{\text{e}}^{{\lambda (l - x_{{\text{t}}} )}} + {\text{e}}^{{ - \lambda (l - x_{{\text{t}}} )}} }}.$$

Thus, we can be obtained, the displacement distribution function of the bolt as:

42 $$s(x) = \left\{ {\begin{array}{*{20}l} {\frac{{F_{{\text{m}}} }}{{k_{{\text{u}}}^{{\prime}} }} \cdot \frac{\cosh [\lambda (l - x)]}{{\cosh [\lambda (l - x_{{\text{t}}} )]}} - \frac{{\overline{C}_{{\text{u}}} }}{{k_{{\text{u}}}^{{\prime}} }}} \hfill & {\quad (x_{{\text{t}}} < x \le l)} \hfill \\ {\frac{{\overline{C}_{{\text{u}}} }}{{2k_{{\text{u}}} }}x^{2} - \frac{{P_{0} }}{{k_{{\text{u}}} }}x + s_{0} } \hfill & {\quad (0 \le x \le x_{{\text{t}}} )} \hfill \\ \end{array} } \right.,$$

the axial force distribution function of the bolt as:

43 $$P(x) = \left\{ {\begin{array}{*{20}l} {\frac{{F_{{\text{m}}} }}{\lambda } \cdot \frac{\sinh [\lambda (l - x)]}{{\cosh [\lambda (l - x_{{\text{t}}} )]}}} \hfill & {\quad (x_{{\text{t}}} < x \le l)} \hfill \\ {P_{0} - \overline{C}_{{\text{u}}} x} \hfill & {\quad (0 \le x \le x_{{\text{t}}} )} \hfill \\ \end{array} } \right.,$$

and the shear stress distribution function of the bolt as:

44 $$\tau (x) = \left\{ {\begin{array}{*{20}l} {\frac{{F_{{\text{m}}} }}{{2{\uppi }r_{{\text{b}}} }} \cdot \frac{\cosh [\lambda (l - x)]}{{\cosh [\lambda (l - x_{{\text{t}}} )]}}} \hfill & {(x_{{\text{t}}} < x \le l)} \hfill \\ {\frac{{\overline{C}_{{\text{u}}} }}{{2{\uppi }r_{{\text{b}}} }}} \hfill & {(0 \le x \le x_{{\text{t}}} )} \hfill \\ \end{array} } \right..$$

According to the continuity of *P*(*x*) at *x* = *x*_t_, the pull-out force at the top of the bolt as:

45 $$P_{0} = \frac{{F_{{\text{m}}} }}{\lambda }\tanh [\lambda (l - x_{{\text{t}}} )] + \overline{C}_{{\text{u}}} x_{{\text{t}}} .$$

In the same way, solving the above equation, *x*_t_ can be obtained.

Similarly, the first-order derivative of the pull-out force function *P*_0_(*x*_t_) at the top of the bolt is obtained as $P_{0}^{{\prime}} (x_{{\text{t}}} ) = \overline{C}_{{\text{u}}} - F_{{\text{m}}} {{\{ 1 - {\text{tanh}}}}^{2} {[}\lambda {(}l{ - }x_{{\text{t}}} {{)]\} }}$. When *x*_t_ increases from 0 to *l*, since $\overline{C}_{{\text{u}}}$ < *F*_m_, $P^{\prime}_{0}$(*x*_t_) first changes from a positive value to 0, and then changes from 0 to a negative value, so it can be seen that *P*_0_(*x*_t_) is a convex function and has a maximum value. By making $P^{\prime}_{0}$(*x*_t_) = 0, the critical failure depth of the shear plane under the ultimate drawing force *P*_0max_ can be obtained as:

46 $$x_{{{\text{tj}}}} = l - \frac{1}{2\lambda }\ln \frac{{1 + \sqrt {1 - \alpha^{{\prime}} } }}{{1 - \sqrt {1 - \alpha^{{\prime}} } }},$$

where $$\alpha^{\prime} = {{\overline{C}_{{\text{u}}} } \mathord{\left/ {\vphantom {{\overline{C}_{{\text{u}}} } {F_{{\text{m}}} }}} \right. \kern-0pt} {F_{{\text{m}}} }}$$.

At this time, the ultimate pull-out force as:

47 $$P_{0\max } = \frac{{F_{{\text{m}}} }}{\lambda }\tanh\left( {\frac{1}{2}\ln \frac{{1 + \sqrt {1 - \alpha^{\prime}} }}{{1 - \sqrt {1 - \alpha^{\prime}} }}} \right) + \overline{C}_{{\text{u}}} \left( {l - \frac{1}{2\lambda }\ln \frac{{1 + \sqrt {1 - \alpha^{\prime}} }}{{1 - \sqrt {1 - \alpha^{\prime}} }}} \right).$$

And, according to the continuity of *s*(*x*) at *x* = *x*_t_, the top displacement of the bolt after the sidewall spring is broken can be obtained as:

48 $$s_{0} = s_{{\text{t}}} - \frac{{\overline{C}_{{\text{u}}} x_{{\text{t}}}^{{2}} }}{{2k_{{\text{u}}} }} + \frac{{P_{0} x_{{\text{t}}} }}{{k_{{\text{u}}} }}.$$

As mentioned earlier, due to the heterogeneity of rocksoil and grout, as well as the interfacial gap caused by changes in temperature and humidity, there are at least two coupling mechanisms of mechanical interlock and friction on the bonding interface of fully grouted bolts. Therefore, the spring-slider model is the one that can best reflect the actual situation among these five load transfer models. Moreover, since the size of *α*′ in this model can reflect different working conditions, the model has a very wide range of applications and is suitable for analyzing all fully grouted bolts.

## Determination of sidewall spring stiffness $k^{\prime}_{{\text{u}}}$

**Figure 17:**
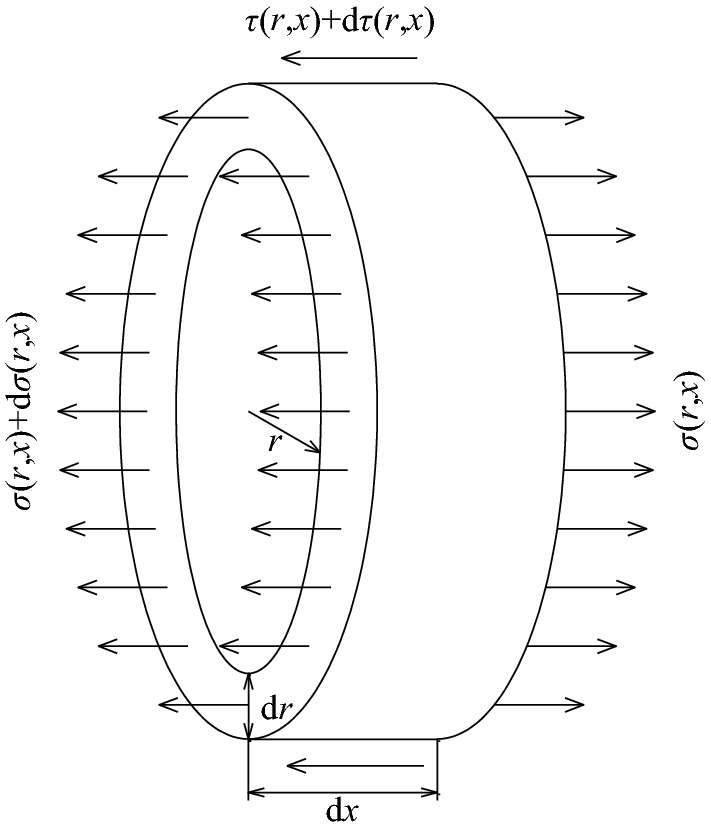
The stress state of the deformed body element around the bolt.

Figure 17 shows the stress state of the infinitesimal element of the deformed body (including grout and rocksoil) around the bolt. The balance equation can be obtained from the force balance condition^33^, as follows:

49 $$r\frac{\partial \sigma (r,x)}{{\partial x}} + r\frac{\partial \tau (r,x)}{{\partial r}} + \tau (r,x) = 0.$$

where, *τ*(*r*,*x*) is the radial shear stress distribution function of the deformed body around the bolt, and *σ*(*r*,*x*) is the normal stress distribution function parallel to the bolt in the deformed body.

During the bolt pulling process, since the variation of *σ*(*r*,*x*) is small, its derivative can be ignored. Substituting the boundary condition $\left. {\tau (r,x)} \right|_{{r = r_{{\text{b}}} }} = \tau (x)$ into Eq. (49) and solving the differential equation, we get:

50 $$\tau (r,x) = \frac{{r_{{\text{b}}} }}{r}\tau (x).$$

where *τ*(*x*) is the shear stress distribution function on the contact surface between the bolt and the grout.

According to the basic principles of elastic mechanics, the following geometric equations can be obtained as:

51 $$\frac{\tau (r,x)}{{G_{{\text{d}}} }} = \frac{\partial u(r,x)}{{\partial x}} + \frac{\partial s(r,x)}{{\partial r}}.$$

In which, *u*(*r*,*x*) is the radial displacement distribution function of the deformable body, *s*(*r*,*x*) is the displacement distribution function parallel to the bolt in the deformable body, and *G*_d_ is the shear modulus of the deformable body.

Similarly, in the process of bolt drawing, the change of radial displacement is much smaller than that of drawing displacement, so the influence of radial displacement can be ignored. Substitute Eq. (50) into Eq. (51), and after integrating, we get:

52 $$s(x) = \frac{{r_{{\text{b}}} \tau (x)}}{{G_{{\text{d}}} }}\int_{{r_{{\text{b}}} }}^{R} {\frac{{{\text{d}}r}}{r}} = \frac{{r_{{\text{b}}} \tau (x)}}{{G_{{\text{d}}} }}\ln \left( {\frac{R}{{r_{{\text{b}}} }}} \right).$$

In the formula, *s*(*x*) is the displacement distribution function of the coordinated deformation on the contact surface of the bolt and the grout, and *R* is the influence radius of the bolt, that is, the radius of the deformation zone.

From Eqs. (15) and (52), it can be known that when the grout has the same characteristics as the rocksoil, there are:

53 $$k_{{\text{u}}}^{{\prime}} = \frac{{2{\uppi }G_{{\text{g}}} }}{{\ln ({R \mathord{\left/ {\vphantom {R {r_{{\text{b}}} }}} \right. \kern-0pt} {r_{{\text{b}}} }})}}.$$

If the properties of the grout are different from those of the rocksoil, Eq. (52) can be rewritten as:

54 $$s(x) = r_{{\text{b}}} \tau (x)\left( {\int_{{r_{{\text{b}}} }}^{{r_{{\text{g}}} }} {\frac{{{\text{d}}r}}{{rG_{{\text{g}}} }}} + \int_{{r_{{\text{g}}} }}^{R} {\frac{{{\text{d}}r}}{{rG_{{\text{r}}} }}} } \right) = r_{{\text{b}}} \tau (x)\left[ {\frac{{\ln ({{r_{{\text{g}}} } \mathord{\left/ {\vphantom {{r_{{\text{g}}} } {r_{{\text{b}}} )}}} \right. \kern-0pt} {r_{{\text{b}}} )}}}}{{G_{{\text{g}}} }} + \frac{{\ln ({R \mathord{\left/ {\vphantom {R {r_{{\text{g}}} )}}} \right. \kern-0pt} {r_{{\text{g}}} )}}}}{{G_{{\text{r}}} }}} \right].$$

In the formula, *G*_g_ is the shear modulus of the grout, *G*_r_ is the shear modulus of the rocksoil, and *r*_g_ is the radius of the borehole.

It can be seen from Eqs. (15) and (54) that when the properties of the grout and the rocksoil are different, there are:

55 $$k_{{\text{u}}}^{{\prime}} = \frac{{2{\uppi }G_{{\text{g}}} G_{{\text{r}}} }}{{G_{{\text{g}}} \ln ({R \mathord{\left/ {\vphantom {R {r_{{\text{g}}} }}} \right. \kern-0pt} {r_{{\text{g}}} }}) + G_{{\text{r}}} \ln ({{r_{{\text{g}}} } \mathord{\left/ {\vphantom {{r_{{\text{g}}} } {r_{b} }}} \right. \kern-0pt} {r_{b} }})}}.$$

## Models verification and discussion

Rong et al.^8^ carried out pull-out tests on smooth steel bolts and threaded steel bolts embedded in concrete block, and simulated the mechanical behavior of the bolt–rock interface. They designed 2 groups of 6 bolts for pull-out test, including 3 threaded steel bolts (test number: 1#, 2#, 3#) and 3 smooth steel bolts (test number: 4#, 5#, 6#). The threaded steel bolts adopts Ø32 Grade II steel (20MnSi), and its mechanical properties are yield strength *σ*_s_ = 390–420 MPa, and tensile strength *σ*_b_ = 560–585 MPa. The smooth steel bolts adopts Ø32 alloy structural steel (40Cr), and its mechanical properties are yield strength *σ*_s_ = 795–800 MPa, and tensile strength *σ*_b_ = 990–995 MPa. The design anchoring length of the two types of bolts is 1.0 m, which are directly cast and buried in the concrete. The concrete grade is R_28_200^#^, and the construction ratio is cement: sand: gravel: water = 1:2.02:3.84:0.46. Ten resistance strain gauges are arranged on the bolt to test the stress values at different depths. The depth of the patch position is 0.05, 0.10, 0.15, 0.20, 0.30, 0.40, 0.50, 0.60, 0.70, 0.80 m, respectively. The maximum design pull-out load is 300 kN, and the load is applied every 50 kN. Take the reading after maintaining 20 min under each level of load. If the bolt yields or the bolt is pulled out of the concrete, the loading will not continue. The bolt displacement is measured with a dial indicator. The pull-out tests for both types of bolts have the same parameters as follows: the bolt length *l* = 1 m; the bolt radius *r*_b_ = 16 mm; the elastic modulus of the bolt is 210 GPa; the drilling radius is the same as the radius of the bolt; the elastic modulus of the concrete base is 26 GPa; and the Poisson's ratio of the concrete is 0.25. In this section, the above models are verified and discussed by applying the pull-out tests of the smooth steel bolt and the threaded steel bolt, respectively. It should be mentioned that, referring to the assumption of Cai et al.^33^, the influence radius of the bolt is taken as *R* = 35*r*_b_, and the *α* in the modified spring model is taken as 0.1.

### Pull-out test of smooth steel bolt

Rong et al.^8^ calculated the bond interface shear strength of the smooth steel bolt based on the test results to be 2.28 MPa, from which it can be seen that the ultimate lateral resistance *F*_m_ is 229kN. According to the test parameters, the ultimate pull-out force *P*_0max_ is calculated, and the result calculated by the spring model is 22.0 kN, the result calculated by the modified spring model is 39.7 kN, and the result calculated by the spring-pulled slider model is 229.0 kN. However, the actual ultimate pull-out force of the test bolt is between 200 and 300 kN. Obviously, only the calculation result of the spring-pulled slider model is the closest to the actual value. According to the derivation of the formula, if the spring-slider model is used to calculate the ultimate pull-out force, the calculated value should be between the calculation results of the spring model and the spring-slider model. Since the $\overline{C}_{{\text{u}}}$ calculated based on the displacement information of the actual test is a negative value, the spring-slider model is also not suitable for simulating this test. Therefore, in this experiment, only the slider model and the spring-pulled slider model can be considered for analysis and calculation. The reason why this happens may be because the bond strength of the bolt interface is too small.

**Figure 18:**
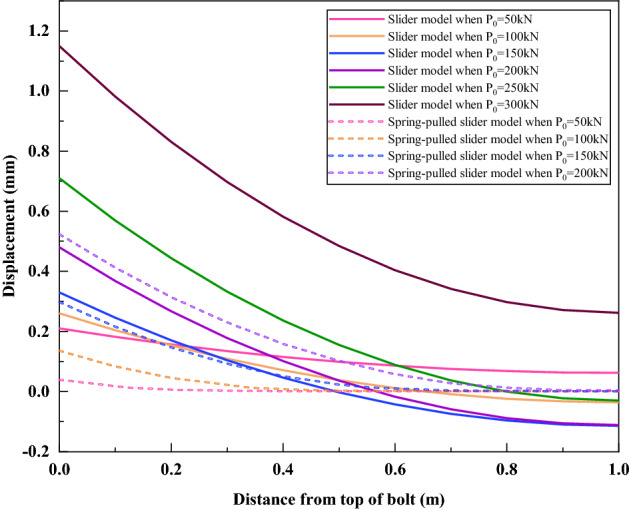
Comparison of displacement distribution curves of slider model and spring-pulled slider model.

Figure 18 compares the displacement distribution curves of the slider model and the spring-pulled slider model along the bolt length under various loads. Obviously, there is a big difference in the curve shape of the two models. Under the same level of load, the displacement distribution curve simulated by the spring-pulled model is slightly gentler than that of the slider model. Since the displacement distribution curve of the slider model needs to use the actual displacement at the top of the bolt for inverse calculation, the displacement at the tail of the bolt is negative under the loads of 100–250 kN, which is obviously inconsistent with the actual situation. This reflects from the side that the displacement of the top of the bolt predicted by the slider model is larger than the actual situation. Under the load of 300 kN, the displacement at the tail of the bolt increased nearly 10 times compared with the previous load, and changed from a negative value to a positive value, indicating that the bolt had been pulled out at this time.

**Figure 19:**
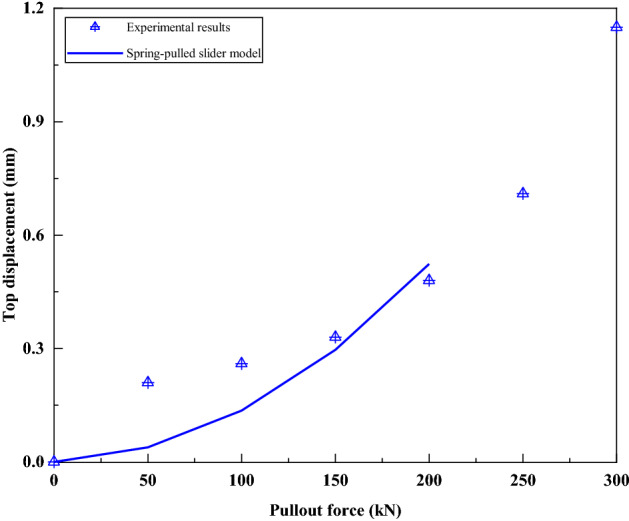
Comparison of bolt top displacement predicted by spring-pulled slider model and test results.

Figure 19 compares the bolt top displacement predicted by the spring-pulled slider model with the test results. Since the ultimate pull-out force calculated by the spring-pulled slider model is 229.0 kN, it is impossible to predict the bolt top displacement under loads of 250–300 kN. It can be seen that although there is some deviation between the two under the loads of 50 kN and 100 kN, the approximate shape of the two is relatively close within the load of 200 kN. This further verifies the applicability of the spring-pulled slider model to simulate such bolts.

**Figure 20:**
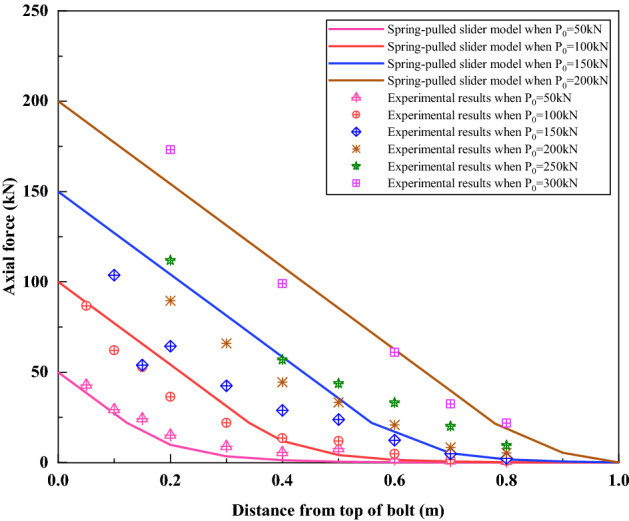
Comparison between the spring-pulled slider model and the test results.

Figure 20 compares the axial force distribution curves simulated by the spring-pulled slider model with the test results. It can be seen that the curves simulated by the spring-pulled slider model are very close to the test results under loads of 50 kN and 100 kN. With the increase of the load, the deviation between the simulation curve and the test results is also increasing, but the general distribution trend of the two is still relatively consistent. Interestingly, the simulated axial force distribution curve under the load of 200 kN is in good agreement with the test results under the load of 300 kN. The reason for this phenomenon may be that the estimated value of the bond strength of the bolt interface is too small. Although the spring-pulled slider model was not particularly ideal for simulating this test, it nonetheless demonstrated the applicability of simulating this type of bolt.

### Pull-out test of threaded steel bolt

Rong et al.^8^ did not give a suggested value for the shear strength of the bond interface of the threaded steel bolt. Ren et al.^2^, Martin et al.^34^, and Ma et al.^35^ used this test to study the mechanical behavior of bolts respectively. The bond interfacial shear strengths given by them are 8.1 MPa, 5.24 MPa and 5.8 MPa, respectively. Combined with the actual test results, the shear strength of the bond interface of the threaded steel bolt suggested in this study is 7.0 MPa, and the ultimate lateral resistance *F*_m_ can be calculated to be 703.7 kN. According to the test parameters, the ultimate pull-out force *P*_0max_ is calculated. The calculated result using the spring model is 67.4 kN, the calculated result using the modified spring model is 122.1 kN, the calculated result using the spring-pulled slider model is 703.7 kN, and the calculated result using the spring-slider model is 458.8 kN. However, due to the low strength of the rebar used in the actual test, the rebar yielded when the load was up to 300 kN, and the load could only be maintained at about 280 kN. According to previous test experience, if the strength of the rebar is large enough, the actual ultimate pull-out force of the test bolt should be between 400 and 500 kN. Apparently only the spring-slider model gives the closest calculation to the actual value. Due to the high shear strength of the bond interface of threaded steel bolts, it is obvious that the slider model is not suitable for analyzing this kind of bolts.

**Figure 21:**
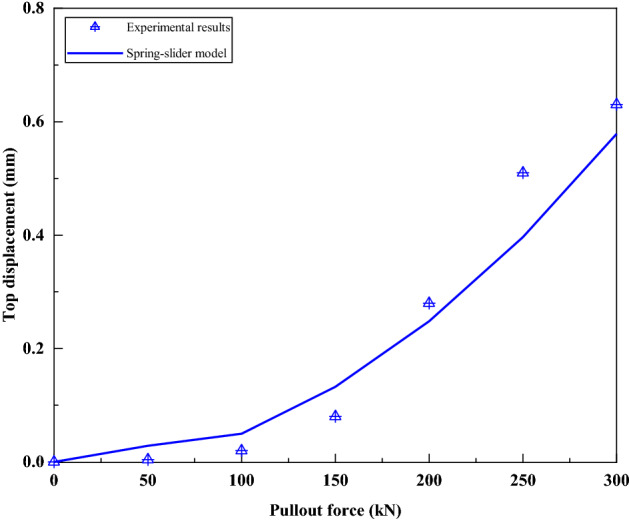
Comparison of bolt top displacement predicted by spring-slider model and test results.

Figure 21 compares the bolt top displacement predicted by the spring-slide model with the test results. It can be seen that there is a good agreement between the two. It also confirms the applicability of the spring-slider model to simulate this kind of bolts.

**Figure 22:**
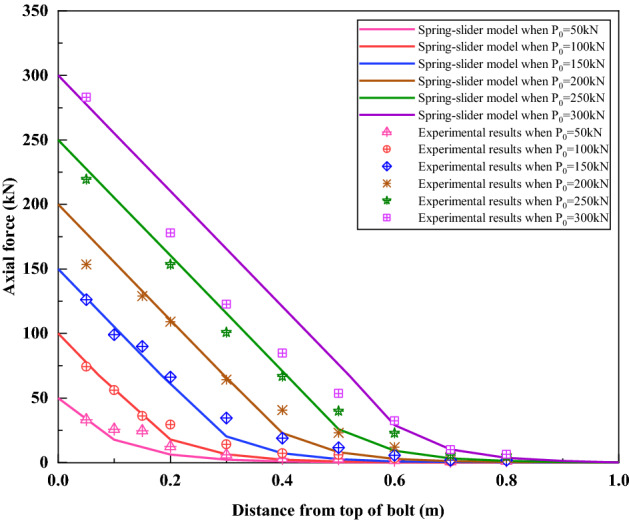
Comparison between the spring-slider model and the test results.

Figure 22 compares the axial force distribution curves simulated by the spring-slider model with the test results. It can be seen from Fig. 22 that under the loads of 50–250 kN, the axial force distribution curve simulated by the spring-slider model is very consistent with the test results. It shows that the spring-slider model has good applicability to simulate this test. Under the load of 300 kN, due to the yielding of the rebar in the actual test, the actual load can only be maintained at 280 kN, which makes some deviations between the simulated axial force distribution curve and the measured results.

By simulating and analyzing the pull-out tests of the two kinds of bolts, it can be seen that the spring-pulled slider model is more suitable for analyzing the smooth steel bolts, while the spring-slider model is more suitable for threaded steel bolts. For different types of bolts, appropriate analysis models should be reasonably selected according to their interface bonding characteristics.

## Parameter analysis

In order to understand the influence of certain parameters on the ultimate pull-out force and shear stress distribution of the bolt, the sensitivity analysis of parameters *α* and *λ* was carried out by using the modified spring model. Other relevant parameters are derived from the pull-out test of the aforementioned threaded steel bolt.

### Influence of parameter *α*

**Figure 23:**
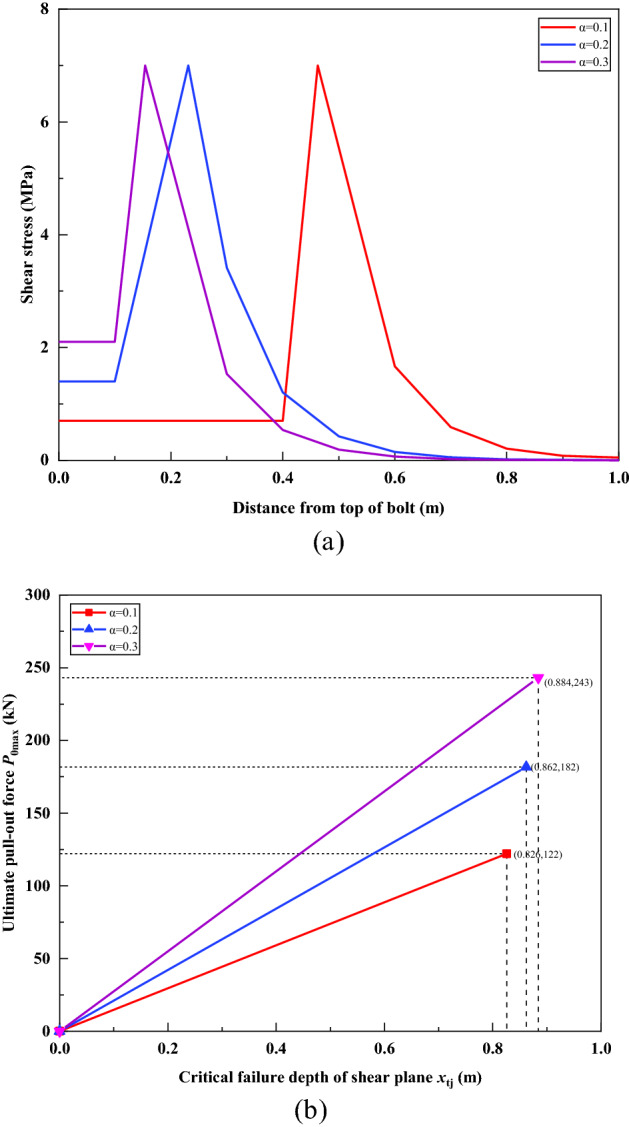
Effect of parameter *α*. (**a**) Under 100 kN load, the influence of different *α* on the distribution of shear stress; (**b**) The influence of different *α* on the ultimate pull-out force of the bolt.

Figure 23 shows the effect of parameter *α* on the analysis results when other parameters remain unchanged. Figure 23a demonstrated the influence of different *α* on the distribution of shear stress under a load of 100 kN. It can be seen that when other parameters remain unchanged, with the increase of *α*, the depth *x*_t_ of the shear failure surface gradually decreases, while the residual frictional resistance *F*_r_ gradually increases. Figure 23b demonstrated the influence of different *α* on the ultimate pull-out force *P*_0max_ of the bolt. With the increase of *α*, the ultimate pull-out force *P*_0max_ of the bolt gradually increases, and the corresponding critical failure depth *x*_tj_ of the shear plane also increases.

### Influence of parameter *λ*

**Figure 24:**
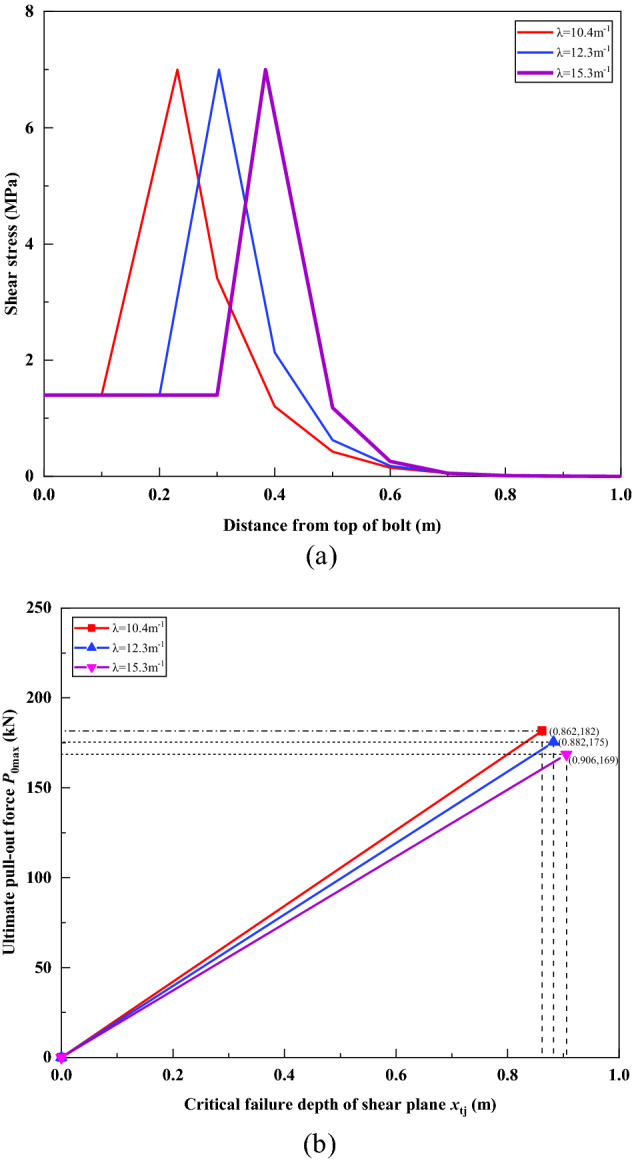
Effect of parameter *λ*. (**a**) When *α* = 0.2, the effect of different *λ* on shear stress distribution under 100 kN load; (**b**) The influence of different *λ* on the ultimate pull-out force of the bolt.

Figure 24 shows the effect of *λ* taking 10.4 m^−1^, 12.3 m^−1^ and 15.3 m^−1^ on the analysis results in the case of α = 0.2. Corresponding to the cases when the elastic modulus of the concrete base is 26 Gpa, 36 Gpa and 56 Gpa, and other parameters remain unchanged. Figure 24a demonstrated the influence of different *λ* on the distribution of shear stress under a load of 100 kN. It can be seen that when other parameters remain unchanged, with the increase of *λ*, the depth *x*_t_ of the shear failure surface is also gradually increasing, while the residual frictional resistance *F*_r_ remains unchanged. Figure 24b demonstrated the influence of different *λ* on the ultimate pull-out force *P*_0max_ of the bolt. With the increase of *λ*, the ultimate pull-out force *P*_0max_ gradually decreases, but the corresponding critical failure depth *x*_tj_ of the shear plane increases. Intuition tells us that when the stiffness $k^{\prime}_{{\text{u}}}$ of the sidewall spring increases, that is, when *λ* increases, the ultimate pull-out force *P*_0max_ of the bolt should increase, and the depth *x*_t_ of the shear failure surface should decrease. This obviously does not match the analysis results in Fig. 24. The reason for the above problem is that when the stiffness $k^{\prime}_{{\text{u}}}$ of the sidewall spring increases, we assume that the ultimate side resistance *F*_m_ remains unchanged, so that the ultimate displacement *s*_t_ of the sidewall spring becomes smaller, and the sidewall spring of the bolt is more easily broken. In fact, there is a certain correspondence between the stiffness $k^{\prime}_{{\text{u}}}$ of the sidewall spring and the ultimate side resistance *F*_m_, and when $k^{\prime}_{{\text{u}}}$ increases, *F*_m_ will generally increase.

The sensitivity analysis of the above key parameters can help us to understand the mechanical behavior of the fully grouted bolt under axial load more deeply.

## Conclusion

Based on the idea of discretization, the analysis method of the bolt spring-element is proposed, and the force of the spring-element is analyzed.

By analyzing the mechanical behavior of the bond interface of the fully grouted bolt, three coupling models of the bond interface are proposed: the slider model, the spring model and the spring-slider model.

Using the spring-element method, five load transfer models including the slider model, the spring model, the modified spring model, the spring-pulled slider model and the spring-slider model are derived respectively. Furthermore, the displacement distribution functions, axial force distribution functions and shear stress distribution functions of the bolt under each model are also derived.

The sidewall spring stiffness $k^{\prime}_{{\text{u}}}$ is analyzed and deduced.

The five models proposed in this study are discussed and verified using the pull-out test of the smooth steel bolt and the threaded steel bolt. It has been verified by experiments that the spring-pulled slider model is more suitable for analyzing the smooth steel bolt, while the spring-slider model is more suitable for the threaded steel bolt. For different types of bolts, a suitable analysis model should be reasonably selected according to their interface bonding characteristics.

Sensitivity analysis of parameters *λ* and *α* was carried out using the modified spring model. Sensitivity analysis of key parameters can help us better understand the mechanical behavior of fully grouted bolts under axial loads.

Analyzing the interfacial mechanical behavior of fully grouted bolts under axial load will help to understand the load transfer mechanism more deeply, so as to guide the design more comprehensively and fully. The five load transfer models proposed in this paper are suitable for different types of bolts under different working conditions. For the same bolt under the same working conditions, the ultimate pull-out force calculated by different load transfer models is also different. Whether the analysis results are close to the actual situation depends on whether the correct and appropriate load transfer model is selected. Therefore, the designers should fully consider the working conditions such as the reinforced object, grouting material, rod material, design bearing capacity, etc., and select an appropriate load transfer model to guide the design when designing a fully grouted bolt. This paper provides important guidance and reference for the design of fully grouted bolts.

## Data Availability

The datasets generated during and/or analysed during the current study are available from the corresponding author on reasonable request.
